# Scintillation Properties of Ba_3_RE(PO_4_)_3_ Single Crystals (RE = Y, La, Lu)

**DOI:** 10.3390/ma16134502

**Published:** 2023-06-21

**Authors:** Yuma Takebuchi, Masanori Koshimizu, Kensei Ichiba, Takumi Kato, Daisuke Nakauchi, Noriaki Kawaguchi, Takayuki Yanagida

**Affiliations:** 1 Division of Materials Science, Nara Institute of Science and Technology (NAIST), Nara 630-0192, Japan; takebuchi.yuma.ty1@ms.naist.jp (Y.T.); ichiba.kensei.if7@ms.naist.jp (K.I.); kato.takumi.ki5@ms.naist.jp (T.K.); nakauchi@ms.naist.jp (D.N.); n-kawaguchi@ms.naist.jp (N.K.); 2 Research Institute of Electronics, Shizuoka University, Shizuoka 432-8011, Japan; koshimizu.masanori@shizuoka.ac.jp

**Keywords:** scintillator, radiation detection, orthophosphate, single crystal

## Abstract

Eulytite-type Ba_3_RE(PO_4_)_3_ (RE = Y, La, and Lu) single crystals were synthesized by the floating zone method, and their scintillation properties were investigated. The powder X-ray diffraction measurement revealed that the single phase of Ba_3_RE(PO_4_)_3_ samples were successfully synthesized. The samples exhibited a luminescence peak due to self-trapped exciton at around 400 nm under vacuum ultraviolet and X-ray irradiation. The X-ray-induced scintillation decay time constants of the samples were several microseconds at room temperature. In the ^241^Am α-ray irradiated pulse height spectra, all the samples showed a clear full energy peak, and the absolute light yields of the Ba_3_Y(PO_4_)_3_, Ba_3_La(PO_4_)_3_, and Ba_3_Lu(PO_4_)_3_ single crystals were estimated to be 960, 1160, and 1220 ph/5.5 MeV-α, with a typical error of ±10%, respectively. The scintillation light yields of the Ba_3_RE(PO_4_)_3_ have been quantitatively clarified for the first time.

## 1. Introduction

Scintillators have a function to convert high-energy ionizing radiation to low-energy photons and are applied in many fields such as medical [1], security [2], well logging [3], and high energy physics [4]. When the scintillator absorbs ionizing radiation, an energetic primary electron is generated from an inner shell. Then, a lot of secondary electrons are produced by Coulomb scattering. The scintillation photons are emitted by electrons recombining at luminescence center. Scintillators are utilized as scintillation detectors by combining with photodetectors. Generally, a scintillator requires several properties, including high scintillation light yield (*LY*); high energy resolution; low afterglow level (*Af*); suitable emission wavelength, which coincides with the spectral sensitivity of the photodetector (e.g., PMT: photomultiplier tube); short decay time; and no deliquescence. Up to now, many researchers have studied novel scintillators [5,6,7,8,9,10,11]. However, there are no scintillators that have all the above requirements, and the development of new scintillators has been still ongoing.

Ba_3_RE(PO_4_)_3_ (BREPO, RE = rare earth) has attracted extensive attention as a luminescent material [12,13,14,15,16,17]. BREPO has high chemical stability, a large band gap, and the ability to accept trivalent lanthanide activators without charge compensation [18,19]. As BREPO belongs to eulytite compounds as well as Bi_4_Ge_3_O_12_ (BGO), which is one of the most popular scintillators [20,21,22,23,24], this material is expected to be a good scintillator. Actually, Shi et al. reported that Ce-doped Ba_3_La(PO_4_)_3_ (BLaPO) powder showed a luminescence intensity of 34% of BGO under X-ray irradiation [25]; as far as we know, this is the only study of the scintillation properties of BREPO. However, the intensity was acquired from a qualitative measurement. Thus, there is room for further investigation of the scintillation properties of BREPO. In addition, as ionizing radiation has higher penetrating power than ultraviolet-visible photons, single crystalline materials having higher transparencies than ceramics or powders are preferable in the scintillator field. However, the investigation of BREPO single crystals were limited in any field thus far [17,26]. If the scintillator had a low transparency at a luminescence wavelength, the number of scintillation photons reaching the photodetector decreases depending on the distance between the emitting point and the photodetector. As a result, the *LY* and the energy resolution of the scintillator are reduced.

In this study, we synthesized Ba_3_Y(PO_4_)_3_ (BYPO), BLaPO, and Ba_3_Lu(PO_4_)_3_ (BLuPO) single crystals. The photoluminescence (PL) and scintillation properties of them were investigated with a quantitative measurement of the scintillation *LY*s as the main purpose. Today, doping of impurity ions to the host material is a common method to develop the novel phosphors, and some researchers neglect the investigation of the properties of the host material itself. However, understanding the properties of the host material is inherently important. For example, the interaction probability between the radiation, materials, and energy migration efficiency basically depends on the host material. Actually, BREPO can accept rare earths other than Y, La, and Lu as well. However, other rare earths act as luminescence centers themselves, and the properties of the materials are often strongly affected by the rare earths. Therefore, we focused on undoped materials containing Y, La, and Lu that had no function as luminescence centers as a start.

## 2. Materials and Methods

The BREPO single crystalline samples were grown by the floating zone (FZ) method. The Y_2_O_3_ (4 N, Furuuchi Chemical, Tokyo Japan), La_2_O_3_ (4 N, Rare Metallic, Tokyo Japan), Lu_2_O_3_ (5 N, Noppon Yttrium, Tokyo Japan), BaCO_3_ (4 N, Rare Metallic), and H_6_NO_4_P (4 N, Sigma Aldrich, St. Louis, MO, USA) powders were used as raw materials. The 10% excess of H_6_NO_4_P was added from stoichiometric ratio to compensate for P evaporation during the crystal growth. The powders were mixed homogeneously by an agate mortar and an agate pestle. The powders were calcined by an electric furnace at 1100 °C, 8 h in air after mixing. Then, the powders were filled into a balloon and hardened by hydrostatic pressure to form into a cylindrical rod. The rods were sintered by an electric furnace at 1200 °C, 8 h in air. The sintering rods were used for the crystal growth by an FZ furnace (Crystal Systems Corporation, FZ-T-12000-X-VPO-PC-YH, Salem, MA, USA). The pulling down and rotation speeds during the crystal growth were 10 mm/h and 10 rpm, respectively. After the crystal growth, a part of the synthesized crystalline rod was cut and mechanically polished by a polishing machine (Byehler, MetaServ 250, Lake Bluff, IL, USA) for measurements, and the remaining parts were crushed into powder for the powder X-ray diffraction (XRD) measurement. A diffractometer (Rigaku, MiniFlex600, Tokyo Japan) with a bias voltage of 40 kV and a tube current of 15 mA was used to measure the powder XRD patterns. The measurement range was from 10 to 80° in 0.02° steps.

A spectrometer (Shimadzu, SolidSpec-3700, Kyoto, Japan) was used for the diffuse transmission spectra measurement. The measurement range was from 200 to 850 nm in 0.5 nm steps. The photoluminescence (PL) excitation and emission spectra were investigated under vacuum ultraviolet (VUV) irradiation by BL-7B beamline in the Ultra Violet Synchrotron Orbital Radiation (UVSOR) synchrotron facility at the Institute of Molecular Science (IMS), Japan. The synchrotron radiation was monochromatized using a 3 m normal-incidence monochromator. The tested wavelength ranges were from 50 to 200 nm in 2 nm steps for the excitation spectra and from 200 to 700 nm in 0.4 nm steps for the emission spectra.

The X-ray-induced scintillation spectra were acquired by our original setup. The sample was attached to the tip of an optical fiber and covered with a Teflon tape, and the sample was irradiated with X-rays. An X-ray generator (Spellman, XRB80N100/CB, Hauppauge, NY, USA) with a tungsten anode target and a beryllium window was used as a radiation source. The bias voltage and the tube current were set to 40 kV and 1.2 mA, respectively. The emitted photons were guided to a spectrometer (Andor, DU-420-BU2 CCD with Shamrock SR 163 monochromator, Belfast England) through the optical fiber. To avoid thermal noise, the CCD was cooled to 188 K by a Peltier module. The X-ray-induced scintillation decay time profiles and the afterglow profiles were studied by an afterglow characterization system. The pulsed LD (for the decay time profiles) or LED (for the afterglow profiles) light sources were the excitation roots. The pulsed width and the repetition frequency were tuned by a digital delay generator (Stanford Research Systems, DG645, Sunnyvale, CA, USA). Visible photons from the LD or LED hit a multi-alkali photocathode (S-20, Sb-Na-K-Cs) of the X-ray tube (Hamamatsu, N5084, Shizuoka, Japan) to convert to electrons. The high voltage bias of 30 kV, supplied by Matsusada HAR-40P0.75 (Shiga Japan), accelerated and led the photoelectrons to a tungsten target to generate bremsstrahlung X-rays. The X-rays were irradiated to the samples through a beryllium window. Emitted photons from the samples were detected by PMT (Hamamatsu, R7400P-06). Once scintillation photons were detected by the PMT, the signal was fed into the photon-counting unit (Hamamatsu, C5594) and then to a PCI-type counting board (Hamamatsu, TSCPC) in a personal computer. The trigger signal was also generated by DG645 and fed into TSCPC. In the case of the afterglow profiles measurement, C5594 and TSCPC were replaced to C9744 and M9003-01, respectively. In order to obtain the scintillation decay time constants, the least-square fitting with two exponential decay functions was utilized. The pulse height spectra with ^241^Am α-ray exposure were measured by below setup. The sample was mounted on the window of the PMT (Hamamatsu, R7600-200) with a silicon grease (OKEN, 6262A, Tokyo Japan) and covered by several layers of Teflon tape to guide emitted photons to the photocathode of the PMT. The scintillation light signal was converted to an electrical signal, and the signal was processed by a pre-amplifier (ORTEC, 113, Oak Ridge, TN, USA), shaping amplifier (ORTEC, 570), and a multichannel analyzer (Amptek, Pocket MCA 8000A, Bedford, MA, USA). To obtain the absolute scintillation *LY*s, a commercial BGO scintillator under ^137^Cs γ-ray irradiation (8000 ph/MeV) was used as a reference. The shaping times were set to 10 and 2 μs for the synthesized BREPO samples and the BGO reference, respectively.

## 3. Results and Discussion

**Figure 1:**
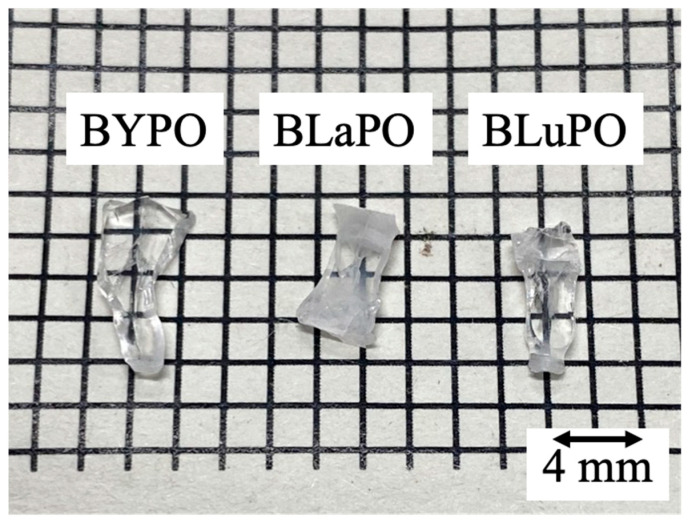
Photo of synthesized BREPO samples after polishing.

Figure 1 shows the photo of the synthesized BREPO samples after cutting and polishing. The size of samples was about 8 mm long × 3 mm wide × 1 mm thick. The colorless transparent crystalline samples were obtained although a part of the samples was opaque and cracked.

**Figure 2:**
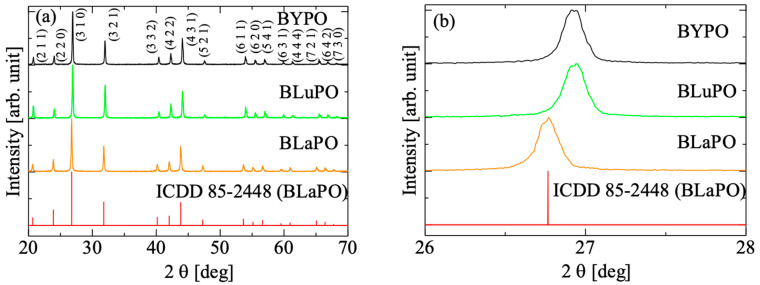
(**a**) XRD patterns of BREPO samples and reference pattern of BLaPO. (**b**) indicates the expansion around 27°.

Figure 2a shows the XRD patterns of the BREPO samples and a reference pattern of BLaPO (ICDD 85-2448), and Figure 2b indicates the expansion around 27°. All the samples showed identical peak pattern of BREPO without impurity phase. The results indicated the samples shown in Figure 1 were single phase of BREPO. From Figure 2b, the peak shift due to the differences of *RE*^3+^ site was observed. The BREPO compounds belonged to a cubic crystal structure with space group of *I*$\bar{4}$*3d*. The unit cells were composed of an isolated PO_4_ tetrahedron and an edge-shared *RE*/Ba octahedron. The diagrams of the lattice structure of BREPO were reported by previous studies [26,27]. The ionic radii of the Y^3+^, La^3+^, and Lu^3+^ ions were 0.900, 1.390, and 0.861 Å, respectively [28]; therefore, the peak shift was consistent with the difference in the ionic radii.

**Figure 3:**
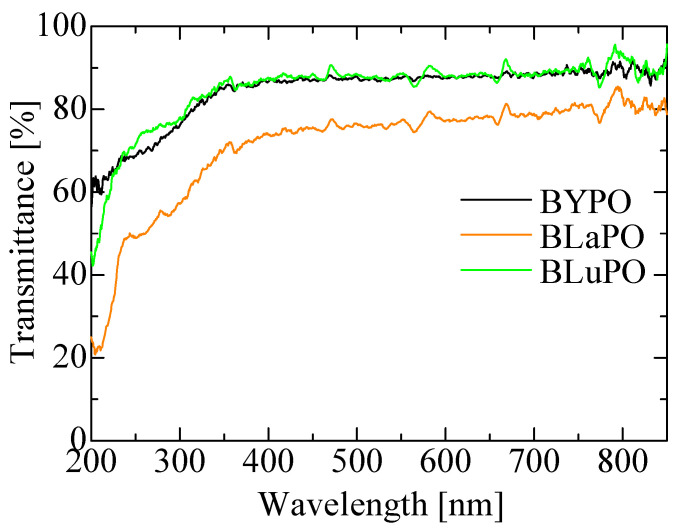
Diffuse transmission spectra of BREPO samples.

Figure 3 shows the diffuse transmission spectra of the BREPO samples. All the samples indicated high transmittance, around 80%, in the visible region. The BLaPO sample showed slightly lower transparency than BYPO and BLuPO samples; it was due to the cracks in the sample. The transmittance gradually decreased from 350 nm; however, the absorption edges of the samples were beyond the measurement limit of the apparatus (200 nm). Therefore, the optical band gaps of the BREPO samples were confirmed to be above 6.2 eV.

**Figure 4:**
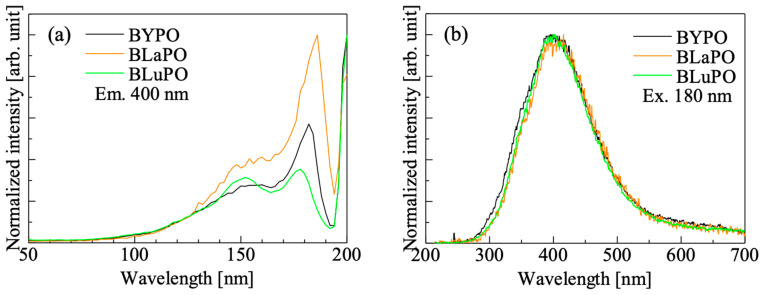
VUV irradiated PL (**a**) excitation and (**b**) emission spectra of BREPO samples.

Figure 4 shows the VUV-irradiated PL (a) excitation and (b) emission spectra of the BREPO samples. The samples showed an excitation peak at 180 nm with a shoulder peak around 150 nm when the monitoring wavelength was set to 400 nm. Similar peaks have been observed in some orthophosphate materials, and the origin of the excitation peak was attributed to the absorption due to PO_4_^3−^ [25,29,30,31]. Under excitation at 180 nm, all the samples showed a broad emission peak around 400 nm. The features of the emission such as emission wavelength, large stokes shift, and broad band width were matched the features of self-trapped exciton (STE) [32], and some phosphates also exhibited luminescence due to excitons at similar wavelengths [33,34,35]. Therefore, the luminescence origin of the BREPO samples was considered to be due to STE.

**Figure 5:**
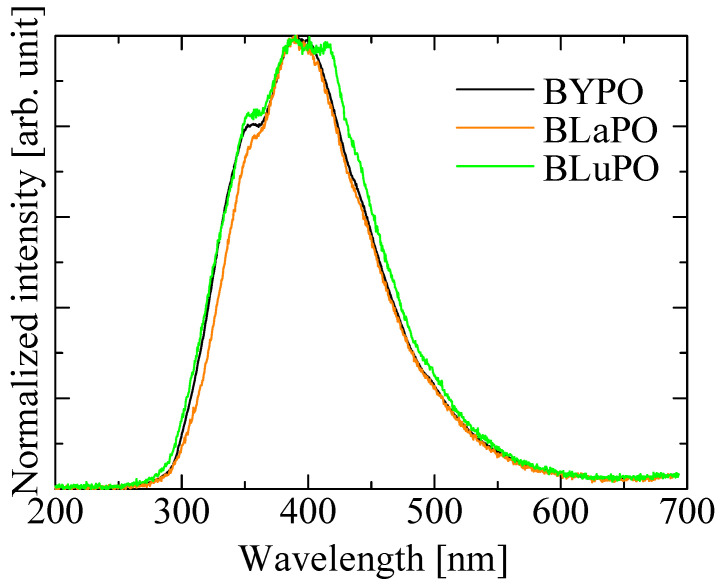
X-ray-induced scintillation spectra of BREPO samples.

Figure 5 shows the X-ray-induced scintillation spectra of the BREPO samples. All the samples exhibited a broad emission band around 400 nm. The emission wavelength and the spectral shape coincided with the luminescence observed in PL emission spectra under VUV irradiation. Therefore, the luminescence origin was ascribed to STE. The luminescence wavelength around 400 nm was suitable for detection by using the typical photodetectors.

**Figure 6:**
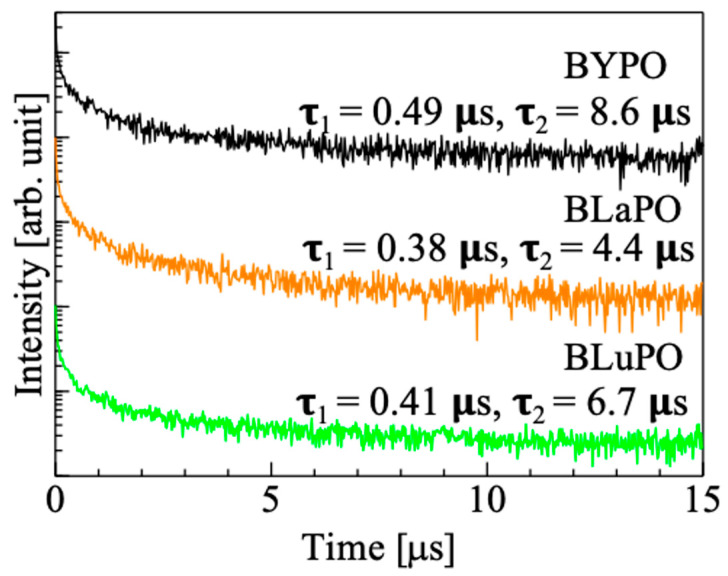
X-ray-induced scintillation decay curves of BREPO samples.

Figure 6 shows the X-ray-induced scintillation decay time profiles of the BREPO samples. Note that the measurement was taken at room temperature. The decay curves of the samples were expressed by the sum of two exponential decay functions. The fast and slow decay time constants were several hundreds of milliseconds and several microseconds, respectively. The fast decay time constant was due to the instrumental response’s function. The slow decay time constant was consistent with that of STE, which was previously reported in some materials at room temperature [36,37]. This result supported that the luminescence observed in Figure 5 was due to STE. However, the accurate measurement of the scintillation decay time profiles was difficult because of low luminescence intensities and high *Af* values (described later) of the samples. Therefore, the discussion about relation or influence of the difference of *RE* site on scintillation decay time constants was difficult in this time.

**Figure 7:**
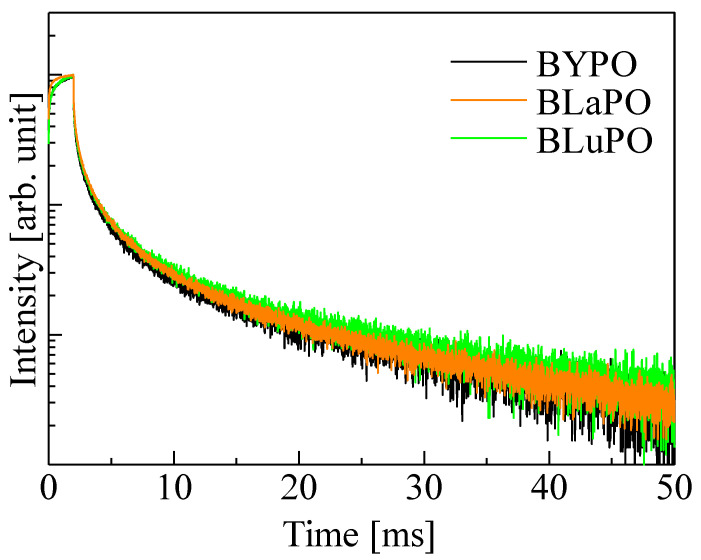
Afterglow profiles of BREPO samples.

Figure 7 shows the afterglow profiles of the BREPO samples. The obtained *Af*s were summarized in Table 1. The *Af* was calculated following formula,

*Af* (%) = 100 × (*I*_2_ − *I*_BG_)/(*I*_1_ − *I*_BG_).

Here, *I*_BG_, *I*_1_, and *I*_2_ are the background signal intensity, the signal intensity during X-ray irradiation, and the signal intensity obtained 20 ms after the irradiation was cut off, respectively. The obtained *Af*s were 1.01, 1.06, and 1.20% for the BYPO, BLaPO, and BLuPO samples, respectively. These *Af*s were quite higher than those of the oxide-based eulytite compounds BGO (10.1 ppm) and Bi_4_Si_3_O_12_ (192.3 ppm) measured by same setup [38,39].

**Figure 8:**
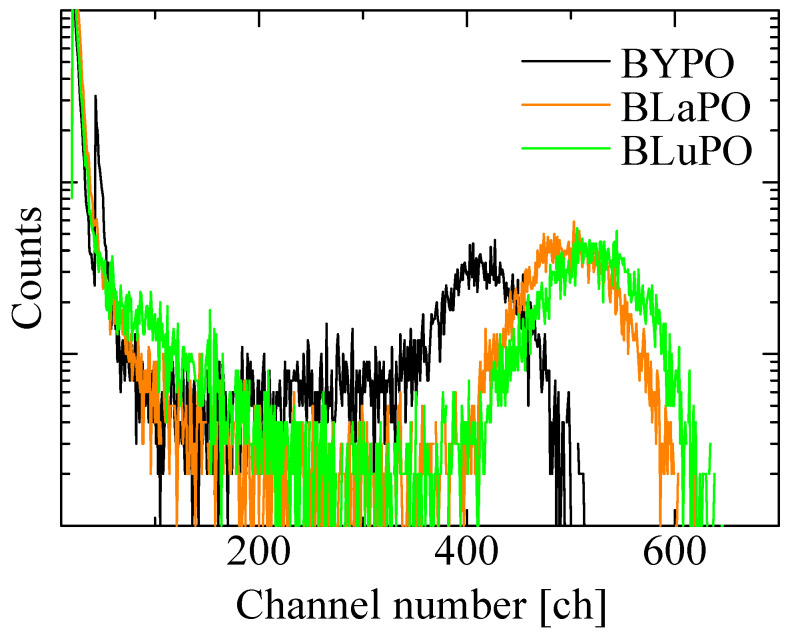
^241^Am α-ray-irradiated pulse height spectra of BREPO samples.

Figure 8 shows the ^241^Am α-ray-irradiated pulse height spectra of the BREPO samples. The obtained *LY*s were summarized in Table 1, as with the *Af*s. All the samples exhibited full energy peaks. The absolute *LY*s of the BYPO, BLaPO, and BLuPO samples were estimated to be 960, 1160, and 1220 ph/5.5 MeV-α, with a typical error of ±10%. The *LY*s of the samples were lower than those of the BGO scintillator. One of the reasons for the low *LY*s was due to the high *Af*s. Afterglow is a type of storage-type luminescence caused by electron captures at some defect centers and re-excitations by weak thermal energy derived from room temperature. A high *Af* means a large number of electrons captured at defect centers; in other words, the energy migration efficiency in the scintillation process is low [40]. As the synthesized B*RE*PO samples had opaque and cracked parts, the amounts of defect centers were considered to be high. Improving crystal quality and reducing defects are required for lower *Af*s and higher *LY*s. In addition, the scintillation decay time constants of several microseconds for the BREPO samples were too long for the pulse height measurement, with a shaping time of 10 μs. This discrepancy led to the underestimation of the *LY*s. Therefore, doping some impurity ions such as the Ce^3+^ ion is interesting to achieve faster decay time constants and higher *LY*s. On the other hand, there was a correlation between *Af*s and *LY*s among the BREPO samples. *LY* depends on not only energy migration efficiency but also quantum efficiency at luminescence centers [41]. Hence, this result implies that the sample containing heavier element possibly had a higher quantum yield of STE. The investigation of quantum yield under VUV excitation is an intriguing topic to further elucidate the scintillation mechanism.

## 4. Conclusions

Eulytite-type BREPO (RE = Y, La, and Lu) single crystals were synthesized by the FZ method, and their optical and scintillation properties were evaluated. The XRD measurement revealed that the synthesized samples were in a single phase of BREPO, although the samples included opaque and cracked parts. All the samples had high transmittances, approximately 80% in the visible region. Under VUV irradiation, the samples showed excitation peaks at 180 nm, with a shoulder peak around 150 nm and an emission peak around 400 nm. The excitation peak was ascribed to the host-related absorption specific to the orthophosphate materials, and the origins of the emission peaks were considered to be due to STE. The luminescence due to STE was also observed in the X-ray-induced scintillation spectra. The luminescence wavelength at around 400 nm was advantageous from the viewpoint of compatibility with the spectral sensitivity of the general photodetectors. The X-ray-induced scintillation decay time constants of the BREPO samples were several microseconds at room temperature. In the pulse height spectra under ^241^Am α-ray irradiation, the *LY*s of the BYPO, BLaPO, and BLuPO samples were estimated to be 960, 1160, and 1220 ph/5.5 MeV-α, with a typical error of ±10%, respectively. The absolute *LY* of BREPO was quantitatively revealed for the first time, although the *LY*s were lower than those of BGO, which also belongs to the eulytite compounds. For further improvement, crystals with fewer amounts of defects are desirable. In addition, the doping of some activators, such as Ce^3+^ ions, is interesting to enhance the *LY*s.

## Figures and Tables

**Table 1:** *Af*s and *LY*s of BREPO samples.

Sample	*Af* (%)	*LY* (ph/5.5 MeV-𝛼)
BYPO	1.01	960
BLaPO	1.06	1160
BLuPO	1.20	1220

## Data Availability

Data will be made available on request.
